# Effectiveness of interventions to screen and manage infections during pregnancy on reducing stillbirths: a review

**DOI:** 10.1186/1471-2458-11-S3-S3

**Published:** 2011-04-13

**Authors:** Sidra Ishaque, Mohammad Yawar Yakoob, Aamer Imdad, Robert L Goldenberg, Thomas P Eisele, Zulfiqar A Bhutta

**Affiliations:** 1 Division of Women and Child Health, The Aga Khan University, Stadium Road, P.O. Box 3500, Karachi-74800, Pakistan; 2 Drexel University College of Medicine, Philadelphia, USA; 3 Department of International Health and Development, Tulane University School of Public Health and Tropical Medicine, New Orleans LA, USA

## Abstract

**Background:**

Infection is a well acknowledged cause of stillbirths and may account for about half of all perinatal deaths today, especially in developing countries. This review presents the impact of interventions targeting various important infections during pregnancy on stillbirth or perinatal mortality.

**Methods:**

We undertook a systematic review including all relevant literature on interventions dealing with infections during pregnancy for assessment of effects on stillbirths or perinatal mortality. The quality of the evidence was assessed using the adapted Grading of Recommendations, Assessment, Development and Evaluation (GRADE) approach by Child Health Epidemiology Reference Group (CHERG). For the outcome of interest, namely stillbirth, we applied the rules developed by CHERG to recommend a final estimate for reduction in stillbirth for input to the Lives Saved Tool (LiST) model.

**Results:**

A total of 25 studies were included in the review. A random-effects meta-analysis of observational studies of detection and treatment of syphilis during pregnancy showed a significant 80% reduction in stillbirths [Relative risk (RR) = 0.20; 95% confidence interval (CI): 0.12 - 0.34) that is recommended for inclusion in the LiST model. Our meta-analysis showed the malaria prevention interventions i.e. intermittent preventive treatment (IPTp) and insecticide-treated mosquito nets (ITNs) can reduce stillbirths by 22%, however results were not statistically significant (RR = 0.78; 95% CI: 0.59 – 1.03). For human immunodeficiency virus infection, a pooled analysis of 6 radomized controlled trials (RCTs) failed to show a statistically significant reduction in stillbirth with the use of antiretroviral in pregnancy compared to placebo (RR = 0.93; 95% CI: 0.45 – 1.92). Similarly, pooled analysis combining four studies for the treatment of bacterial vaginosis (3 for oral and 1 for vaginal antibiotic) failed to yield a significant impact on perinatal mortality (OR = 0.88; 95% CI: 0.50 – 1.55).

**Conclusions:**

The clearest evidence of impact in stillbirth reduction was found for adequate prevention and treatment of syphilis infection and possibly malaria. At present, large gaps exist in the growing list of stillbirth risk factors, especially those that are infection related. Potential causes of stillbirths including HIV and TORCH infections need to be investigated further to help establish the role of prevention/treatment and its subsequent impact on stillbirth reduction.

## Background

Stillbirth, defined as a newborn having no sign of life at delivery, is one of the most common adverse outcomes of pregnancy. About 3.2 million stillbirths occur worldwide each year [1]. The highest overall figures of stillbirths have been reported from countries in South-East Asia and sub-Saharan Africa [1]. Globally, infection is a leading cause of stillbirth and accounts for an estimated half of all stillbirths today, especially in developing countries [2]. A notable association exists between infection and gestational age. A review reported that the earlier the fetal death during gestation, the more likely it is to be caused by an infection [3]. For example, in one study, 19% of fetal deaths less than 28 weeks were associated with an infection, while only 2% of term stillbirths were infection-related [4]. Direct infection, placental damage, and severe maternal illness are the most commonly reported mechanisms by which infections may cause stillbirths [5].

Among infections, various organisms have been implicated as causing stillbirth, including bacteria, viruses, protozoa, helminthes and fungi [6]. Syphilis remains an avoidable cause of infection related stillbirths Plasmodium *falciparum* malaria has been associated with stillbirth especially in primigravidas owing to its high prevalence and extensive placental damage [7]. TORCH infections, which include Toxoplasmosis, Other (syphilis, varicella-zoster, parvovirus B19), Rubella, Cytomegalovirus (CMV), and Herpes infections, are infections during pregnancy that are associated with congenital anomalies and possibly stillbirths [8,9]. Most of the TORCH infections cause mild maternal morbidity, but have serious fetal consequences [8]. Periodontal disease is another important chronic infectious disease of humans which is commonly present in pregnancy[10]. It’s prevalence range from 35 to 100% (especially gingivitis) during pregnancy [11]. The risk of adverse pregnancy outcomes including preterm birth, fetal growth restriction may be increased in women with periodontal disease [12-15]. This may be the result of systemic inflammatory responses due to a persistent periodontal infection, or periodontal disease may be a marker for infection elsewhere in the body [12,16]. Helminthiasis is infestation of the human body by parasitic worms [17]. Millions of pregnant women globally are infected with intestinal nematodes [18] which leads to chronic blood loss, and can be a major cause of iron deficiency anemia in reproductive age, which is a risk factor for adverse perinatal outcome [19]. A more detailed description of background literature on maternal infections during pregnancy and their association with stillbirths is given in Additional File 1.

The objective of this review was to synthesize up-to-date evidence on the role of diagnosis and management of infections in pregnancy and their effects on stillbirth. This paper is a part of series of papers reviewing the effect of interventions to be included in the Live Saved Tool (LiST) [20]. This process involves qualitative assessment of available evidence according to the Grading of Recommendations, Assessment, Development and Evaluation (GRADE) approach and quantitative analysis based on rules developed by the Child Health Epidemiology Reference Group (CHERG) [20,21]. More details about CHERG methods and LiST are available in the methods section of this paper and the CHERG methods paper [20].

## Methods

### Literature search

We searched all relevant literature on interventions dealing with infections during pregnancy to quantify their impact on stillbirths and perinatal mortality. The last date of the search was February 1^st^ 2010. The following infections were included in this review: syphilis, *P. falciparum* malaria, HIV, bacterial vaginosis, ascending bacterial infections (chorioamnionitis), asymptomatic bacteriuria, periodontal disease, helminthiasis, and TORCH infections. The databases searched included PubMed, the Cochrane Library, The Lancet Series and hand search of bibliographies of relevant reviews. Experts were also contacted in the field for unpublished data. The search strategy for different infections is given in Additional File 2:

### Selection (inclusion/exclusion criteria)

The study designs primarily considered were randomized and quasi-randomized trials however observational studies were also considered where data were not available from randomized trials. There was no restriction on language or publication status. There were no limits on gestational age at the time of enrolment in the study. For IPTp (intermittent preventive treatment of P. Falciparum), we sought studies that measured the effect of at least 2-doses per pregnancy of IPTp with sulfadoxine-pyrimethamine versus a placebo, independent of Insecticide treated nets (ITNs). Trials that compared 2-doses of IPTp to another drug (not a placebo) were excluded, as were trials that assessed IPTp compared to ITNs where the effect of IPTp against a placebo could not be isolated. For ITNs, we sought studies that measured the effect of access to ITNs among pregnant women versus no ITNs on stillbirths. Trials that compared ITNs to untreated nets were excluded, as were trials that assessed ITNs plus IPTp where the effect of ITNs against no mosquito net (placebo) could not be isolated.

### Data abstraction and validity assessment

Each study that satisfied the eligibility criteria was included in the review. A standardized data extraction sheet was used [20]. The data were abstracted for key variables like; participants’ characteristics, sample size, location, study design and limitations, description of intervention and control groups.

The individual studies were graded according to CHERG adaptation of GRADE criteria [20]. A study was graded as that of ‘high’ ‘moderate’ ‘low’ or ‘very low’ quality based on strengths and limitations of the study. Studies received an initial score of high if they were a randomized or cluster randomized trial. The grade was decreased one grade for each study design limitation. In addition, studies reporting an intent-to-treat analysis or with statistically significant strong levels of association (>80% reduction) received 0.5-1 grade increases. Any study with a final grade of very low was excluded. The overall quality of evidence of an outcome was also assessed and graded according to the CHERG adaptation of the GRADE technique [20]. This assessment was based on three components: 1) the volume and consistency of the evidence; 2) the size of the effect, or risk ratio; and 3) the strength of the statistical evidence for an association between the intervention and outcome, as reflected by the p-value [20,21].

### Quantitative data synthesis

We performed meta-analyses where data were available from more than one study for an outcome. The primary outcomes of interest were stillbirths and perinatal mortality. The reason for including perinatal mortality as an outcome was based on the fact most of the studies do not report disaggregated data for stillbirths but do that by combining stillbirths with early neonatal deaths (i.e. perinatal mortality). This exercise is in accordance with the basics of CHERG rules which take into account the biological plausibility of the intervention and limitations of measured outcomes [20]. For example studies may not collect the outcome of interest (e.g., pneumonia mortality) but may collect data on severity of disease (e.g. hospital admission). CHERG rules will thus consider a range of outcomes (where necessary) and choose a point estimate which is the most conservative and represent the expected effect of the intervention based on biological plausibility [20].

We used dichotomous values for pooling the data, except for insecticide-treated mosquito nets (ITNs) where data were pooled by generic inverse variance method of meta-analysis. The summary estimates were described as relative risk (RR) or Odd ratios (OR) with 95 % confidence interval (CI). The assessment of statistical heterogeneity among trials pooled data was done by visual inspection (i.e. the overlap of the confidence intervals among the studies), Chi square (P-value) and I^2^ values. An I^2^ value greater than 50% was taken to represent substantial heterogeneity in the pooled data. In case of substantial heterogeneity, causes were explored by sensitivity analysis. A random effects model was used for the meta-analysis in case of substantial heterogeneity. All the analyses were conducted using Review Manager 5 [22]. We applied the CHERG Rules for Evidence Review to recommend a final estimate for reduction in stillbirth from interventions for a specific maternal infection during pregnancy [20].

## Results

**Figure 1:**
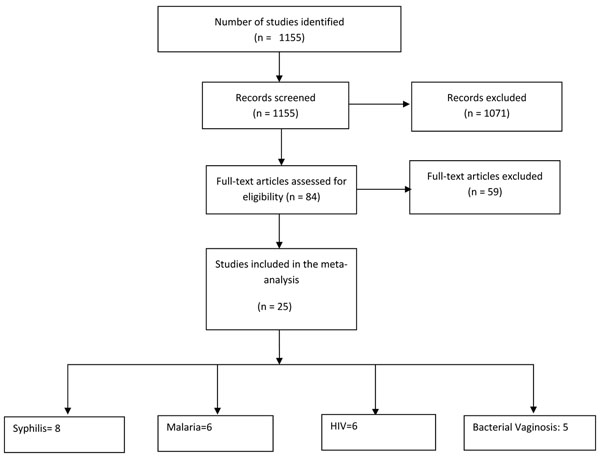
Flow diagram showing identification of studies

A total of 1155 hits were identified from our search strategies (Figure 1). After screening the titles and abstracts, 84 studies were initially considered eligible. We thoroughly reviewed the abstracts and full texts, where available and 25 studies were selected for inclusion in the meta-analyses (Additional file 3). Additional File 4 outlines the characteristics of included studies.

### Syphilis

No randomized trials were found for treatment of syphilis and its effect on prevention on stillbirths. However, a random-effects meta-analysis of 8 observation studies by Blencowe et al. in this supplement has shown an estimated reduction of 80 % (RR 0.20, 95% CI 0.12 - 0.34) in syphilis related stillbirths with use of penicillin [23]. It is however important to note that none of the included studies in this meta-analysis made any attempt to control for systematic differences between treated and untreated women (confounding). For example women not attending antenatal clinic and/ or not complying with complex penicillin treatment regimens may differ in their risk profiles for stillbirth, preterm delivery and neonatal death from fully compliant infected women.

### Malaria

**Figure 2:**
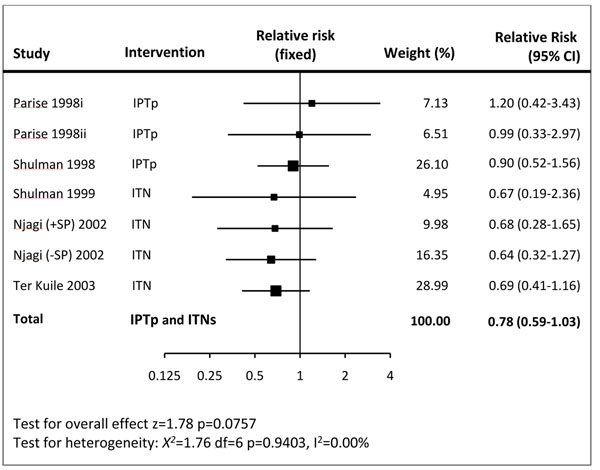
Forest plot for meta-analysis of the effect of malaria prevention in pregnancy (IPTp and ITNs) used during first or second pregnancy on preventing stillbirths

There were 6 randomized controlled trials that addressed prevention of malaria during pregnancy by IPTp or ITN. As the effects of IPTp and ITNs used during pregnancy have similar causal pathways for preventing stillbirth, and as there is no evidence of a synergistic effect between them [24], the data were pooled irrespective of method used. The combined results show 22% reduction in stillbirths (RR = 0.78; 95% CI: 0.59 – 1.03) (Figure 2). A subgroup analysis of three trials [25-27] that evaluated the impact of IPTp with sulfadoxine-pyrimethamine vs. placebo showed no effect on stillbirth (RR = 0.96; 95% CI: 0.62 – 1.50; fixed model) or perinatal mortality (RR = 0.78; 95% CI: 0.52 – 1.17; fixed model). Pooled results of three studies that used ITNs in pregnancy yielded a statistically significant association with reduced fetal loss (RR 0.67, 95% CI 0.47 to 0.97) compared to controls.

### HIV

**Figure 3:**
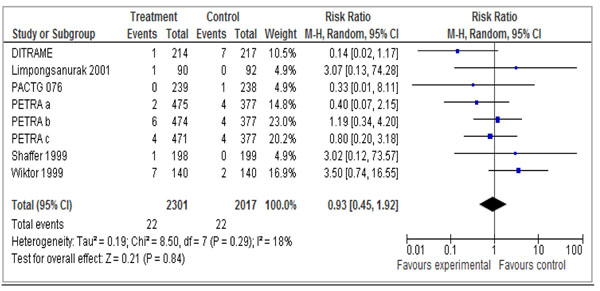
Treatment of HIV in pregnancy: *Any antiretroviral vs. placebo* Outcome: Stillbirth

Our pooled meta-analysis of 6 studies [28-33] failed to show a statistically significant reduction in stillbirth when the use of anti-retrovirals in pregnancy was compared to control (RR 0.93 95% CI 0.45 – 1.92) (See Figure 3).

### Bacterial vaginosis

**Figure 4:**
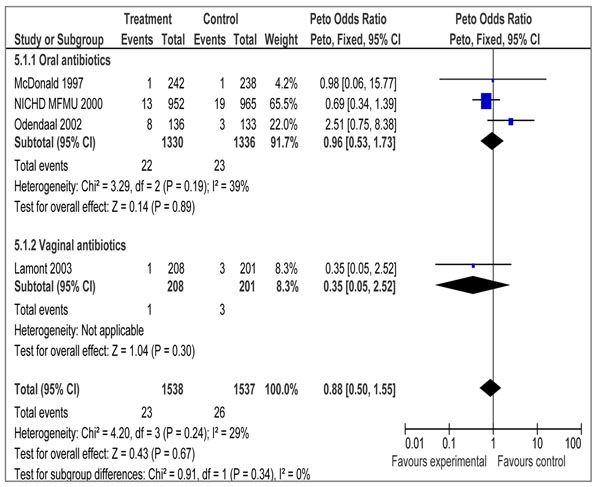
Treatment of bacterial vaginosis in pregnancy: *Any antibiotic versus placebo/no* Outcome: Perinatal death

Our pooled analysis combining four studies [34-37] showed a non-significant impact of treatment of bacterial vaginosis on perinatal mortality (OR 0.88 95% CI 0.50 – 1.55). Sub-group analysis showed similar results with the use of oral (OR 0.96 95% CI 0.53 – 1.73) and vaginal (OR 0.35 95% CI 0.05 – 2.52) antibiotics (Fig 4). There are no data on Still Births.

### Chorioamnionitis

The Cochrane review by Kenyon et al. [38] studies the use of antibiotics for preterm premature rupture of membrane (pPROM) and reports a statistically significant 43% reduction in risk of chorioamnionitis (RR = 0.57; 95% CI: 0.37 – 0.86). There was however no impact on perinatal mortality/or fetal death before discharge (RR = 0.90; 95% CI: 0.74 – 1.10). Another Cochrane review by Flenady and King [39] on antibiotics for PROM at or near term showed no impact of antibiotics on chorioamnionitis (RR = 0.60; 95% CI: 0.30 – 1.18) nor on perinatal mortality (RR = 0.98; 95% CI: 0.14 – 6.89). There were no separate data on stillbirth.

### Asymptomatic bacteriuria

Studies have looked at the association between asymptomatic bacteriuria during pregnancy and perinatal mortality. In a study performed from 1972 to 1979, pregnant women with asymptomatic bacteriuria had a significantly higher perinatal mortality (64.9 vs. 15.6, p < 0.001) [40]. However, a more recent population-based retrospective study by Sheiner et al. showed that patients with asymptomatic bacteriuria, in whom antibiotic treatment was recommended, had similar perinatal mortality rates compared to women without asymptomatic bacteriuria (1.5% vs. 1.4%, P = 0.707) [41]

### Periodontal disease

While published data convincingly display a link between periodontal disease and stillbirth incidence [10,15,42-44], there is limited literature on interventions for periodontal disease and impact on stillbirths. The systematic review by Polyzos et al. [14] studied the impact of periodontal care during pregnancy and found a non-significant effect on abortions and stillbirths (OR = 0.73; 95% CI: 0.41 to 1.31). In a recent randomized controlled trial, 1082 pregnant women with periodontal disease were allocated either to receive periodontal treatment in mid-pregnancy or after delivery. There were four unexplained stillbirths in the control group and no losses in the treated group, but the results were non-significant (P = 0.12) [45]. A meta-analysis on periodontal care during pregnancy versus treatment after birth based on two studies [13,45] has shown a 70% significant reduction in stillbirth (RR = 0.30; 95% CI: 0.12 – 0.76) (Philippa Middleton, personal communication).

### Helminthiasis

A recent study from Uganda on deworming during pregnancy has shown no effect of treatment with albendazole or praziquantel on incidence of stillbirths compared to placebo [46]. Another open label, randomized controlled trial from Uganda that compared ivermectin, albendazole, combined ivermectin/albendazole compared with placebo showed similar results (P = 0.13) [47]. The Cochrane review by Haider et al. reported the pooled effect of use of anti-helminthic therapy on perinatal mortality based on two RCTs [48,49] and showed no overall effect (RR = 1.10; 95% CI: 0.55 – 2.22).

#### *TORCH INFECTION*: TOXOPLASMOSIS, OTHER (SYPHILIS, VARICELLA-ZOSTER, PARVOVIRUS B19), RUBELLA, CYTOMEGALOVIRUS (CMV), AND HERPES INFECTIONS

A case control study from Jordan reports that the presence of Toxoplasmosis *gondii* was significantly higher in those with adverse pregnancy outcomes compared to controls (54% vs. 12%, p<0.02) [50]

*Maternal rubella* has been implicated as a cause of stillbirths [51]. In Guinea-Bissau, stillbirth rates were increased 4- to 9-fold if the mother was infected with rubella during her pregnancy [3].

### Cytomegalovirus

A study from Australia [52] reported that 9% of blood samples taken from stillbirths by cardiac puncture were PCR positive for CMV. A recent study from Greece using PCR, showed significantly increased levels of CMV (16%) in the placentas of stillbirths compared to controls (3%) [53].

*Herpes simplex* infections have also been described as a cause of fetal death [54]. However, Herpes simplex viruses rarely, if ever, cause stillbirth, likely because the virus rarely causes an intrauterine infection. Neonatal infections are acquired during fetal passage through an infected birth canal. There is a paucity of evidence with regards to the significance of Herpes virus in causing stillbirths. Also, whether treatment and prevention have a significant positive impact on adverse pregnancy outcomes remains elusive.

Tables 1 - 4 outline the quality grading of the overall evidence of all the interventions considered in this review and described below in the same order.

**Table 1:** Quality assessment of trials of interventions for infections (syphilis) in pregnancy

	Quality Assessment					Summary of Findings		
				**Directness**		**No of events**		

**No of studies (ref)**	**Design**	**Limitations**	**Consistency**	**Generalizability to population of interest**	**Generalizability to intervention of interest**	**Intervention**	**Control**	**Relative Risk (95% CI)**

***Syphilis and stillbirth: LOW*** **outcome specific quality**								

8	Observational studies	none	Some heterogeneity and 5 out of 8 studies show benefit	Yes	Yes	Not available	Not available	0.20 (0.12 - 0.34)

**Table 2:** Quality assessment of trials of the evidence for prevention in malaria in pregnancy (IPTp and ITNs) used during first or second pregnancy for preventing stillbirths

Quality assessment						Summary findings				
				**Directness**		**Intervention**		**Control**		

**No of studies**	**Design**	**Limitations**	**Consistency of results**	**Generalizability to population of interest**	**Generalizability to intervention of interest**	**Number of events**	**Denominator**	**Number of events**	**Denominator**	**RR (95% CI)**

***IPTp for preventing stillbirths:*** **High outcome-specific quality**										

3	RCT		Consistent *X^2^* for heterogeneity (2 df) = 0.23	High	High	44	1,489	36	1,083	0.96 (0.62-1.50)

***ITNs for preventing stillbirths:*** **High outcome-specific quality**										

4	CRCT	Not blinded	Consistent *X^2^* for heterogeneity (3 df) = 0.03	High	High	Unknown	Unknown	Unknown	Unknown	0.67 (0.47-0.97)

***Effect of IPTp and ITNs combined on stillbirths :*** **Moderate outcome-specific quality**										

7	RCT/ CRCT		Consistent *X^2^* for heterogeneity (6 df) = 1.76	High	High	Unknown	Unknown	Unknown	Unknown	0.78 (0.59-1.03)

RCT: Randomized controlled trial: CRCT: Community randomized controlled trial: RR: Relative risk: CI: Confidence Interval

**Table 3:** Quality assessment of trials of interventions for infections (HIV) in pregnancy

Quality Assessment						Summary of Findings		
				**Directness**		**No of events**		

**No of studies (ref)**	**Design**	**Limitations**	**Consistency**	**Generalizability to population of interest**	**Generalizability to intervention of interest**	**Intervention**	**Control**	**Relative Risk (95% CI)**

***Any anti-retroviral use for HIV and stillbirth: MODERATE*** **outcome specific quality**								

6	RCTs	none	Some heterogeneity from meta-analysis; 4 out of 8 data sets showing benefit	Yes	Yes	22	22	RR (fixed) = 0.93 (0.45 - 1.92)

**Table 4:** Quality assessment of trials of interventions for infections (bacterial vaginosis) in pregnancy

Quality Assessment						Summary of Findings		
				**Directness**		**No of events**		

**No of studies (ref)**	**Design**	**Limitations**	**Consistency**	**Generalizability to population of interest**	**Generalizability to intervention of interest**	**Intervention**	**Control**	**Odd Ratios (95% CI)**

***Antibiotics for bacterial vaginosis and perinatal death: MODERATE*** **outcome specific quality**								

4	RCTs	Low recruitment response in one study, also allocation concealment unclear	Some heterogeneity in meta-analysis; 3/4 studies show benefit	Yes	Yes	23	26	OR = 0.88 (0.50 - 1.55)

## Discussion

Syphilis remains a major cause of avoidable perinatal death in many countries despite being treatable, and despite the WHO recommendation that all pregnant women be tested as part of routine antenatal care. Most studies report syphilis to have a relative risk of stillbirth in the range of 2 to 5; however, a Tanzanian study reported a relative risk of 18 for women with active syphilis [55]. In some areas of sub-Saharan Africa, about 25% to 50% of all stillbirths were associated with syphilis [56]. Syphilis also contributes to stillbirths in other areas of the world including Russia, Asia and South America [57]. The findings of a meta-analysis by Blencowe et al. correlate with the above observations and shows that treatment of syphilis during pregnancy can significantly reduce syphilis related stillbirths by up to 80 % [23]. This estimate has been recommended for inclusion in the LiST model with a quality grade of ‘low’ (Table 1). The overall evidence was graded as ‘low’ as all the included studies in the meta-aalysis were observational studies.

Malaria in pregnancy is an important preventable cause of maternal and perinatal morbidity and mortality [7]. The WHO advocates a three-pronged approach to control malaria in pregnancy that includes the use of ITNs, IPTp and case management of parasitemia with artemisin-based therapies, especially in areas where malaria is endemic [58]. Our meta-analysis suggests that malaria prevention interventions in pregnancy (IPTp or ITNs) can reduce stillbirths by 22% in malaria endemic countries (RR = 0.78; 95% CI: 0.59 – 1.03). While this effect is only marginally significant (and thus graded as that of ‘moderate’ quality in table 4), there is other evidence to suggest that such interventions could be highly effective in improving maternal and birth outcomes [58-61]. This is supported by the fact that IPTp has been shown to significantly reduce placental malaria, as well as maternal anemia [60], which are associated with adverse pregnancy outcome.

The pandemic of human immunodeficiency virus (HIV) in pregnancy is one of the major health problems today [63]. Maternal HIV infection may increase the risk of stillbirth [64]. A study from Zambia has reported an inverse relationship between decreasing CD4 cell counts in HIV seropositive women and stillbirth (P =0.000) [65]. Our pooled analysis however failed to show a statistically significant reduction in stillbirth when antiretrovirals for pregnant women were compared to placebo (RR = 0.93; 95% CI 0.45 – 1.92) and thus no recommendations have been made for LiST regarding this intervention. Results of our review are consistent with the results from a Cochrane Review by Volmink et al[66]. Data was not pooled in this Cochrane review however none of the six included trials showed a significant impact of antiretrovirals in pregnancy on stillbirth[66]. Pooled analysis from a review by Suksomboon et al. [67], that included five randomized trials, showed a non-significant impact on stillbirth (RR=1·11 95% CI 95% 0·48- 2_·_56).

Bacterial vaginosis in pregnant women is common, ranging from 14% to 21% in Western countries and 13.6–18% in Asian countries [68]. However, very few perinatal deaths have been reported in relation to maternal bacterial vaginosis [68]. Mc Donald reports a non-significant association of the treatment of bacterial vaginosis in pregnancy with reduction in perinatal mortality [34]. Our meta-analysis of four studies (3 for oral and 1 for vaginal antibiotic) failed to yield a significant impact on perinatal mortality (Figure 4). Whether treatment of bacterial vaginosis will reduce stillbirths is unknown and would need to be validated by future trials. Applying the CHERG Rules for Evidence Review, studies included for bacterial vaginosis and perinatal death were ranked as having a ‘moderate’ grade of evidence. Included studies had limitations, including a low recruitment response and unclear allocation concealment. The evidence to date does not suggest any benefit of screening and treating all pregnant women for asymptomatic BV to prevent stillbirths. Although antibiotic treatment targeting bacterial vaginosis may be of value in some women [37], further research needs to be carried out to study the potential impact of antibiotics on chorioamnionitis, which is a well-established risk factor for stillbirth [6].

There are very few RCTs that have looked at the impact of periodontal care on stillbirth or perinatal mortality, with most trials reporting preterm birth as an outcome. A recent meta-analysis of periodontal care suggests that there may be an impact on stillbirths (Middleton P, 2010 personal communication), but needs confirmation in further trials from developing countries. This is an emerging area of research that needs more studies.

There is very limited evidence of the impact of anti-helminthic therapy on stillbirths. There has been a tendency towards increased risk of stillbirths and perinatal mortality that is non-significant based on RCTs [17]. Maternal deworming, however, does hold promise for improving the health of pregnant women in endemic areas, particularly in terms of alleviating risk of anemia and subsequently the risk of adverse pregnancy outcomes [18]. It is, therefore, recommended in specific, situational contexts such as where soil associated helminthiasis rates are high and iron-deficiency anemia rates endemic [69].

TORCH infection is an important risk factor for stillbirths [8]. However, the pathways which lead from infectious risk factors to fetal death are, for the most part, not well defined [70,71]. There is a dearth of good quality evidence on association of treatment of TORCH on stillbirth and we recommend further research to study the associations of these infections with stillbirth and perinatal mortality.

The review employs a comprehensive search strategy thus increasing the chance of retrieving all relevant studies. Mainly randomized controlled and quasi randomized trials have been considered that provide a high quality level of evidence. It is however important to note that data from interventional studies were scarce. A high quality research agenda addressing the contribution of infections to stillbirth, especially in developing countries, is needed to reduce stillbirths worldwide. At present, large gaps exist in the growing list of stillbirth risk factors, especially those that are infection related [72]. The clearest evidence of impact on stillbirth prevention is adequate prevention and treatment of infections such as syphilis and possibly malaria. Other potential causes of stillbirth including HIV, bacterial vaginosis, ascending infections and TORCH infections need to be investigated further to help establish the role of prevention/treatment and its subsequent impact on stillbirth reduction [72]. Efficacious interventions exist for certain maternal infections and conditions for which the evidence of plausible benefit is not very clear. Therefore, efforts need to be geared to conduct high quality trials for us to ascertain the full extent of the relation between interventions and their potential to reduce stillbirths.

## Conclusions

The clearest evidence of impact in stillbirth reduction is adequate prevention and treatment of maternal infections such as syphilis and possibly malaria. At present, large gaps exist in the growing list of stillbirth risk factors, especially those that are infection related.

### Key Messages

A reduction of 80% is observed in the incidence of stillbirths in pregnant women with syphilis receiving penicillin.

Malaria prevention intervention in pregnancy through IPTp and ITNs providemay reduce stillbirths by 22% during the first or second pregnancy in areas of stable *P. falciparum* malaria transmission.

More studies need to be conducted to determine the effectiveness of interventions for diseases such as HIV, ascending bacterial infections and TORCH infections for reducing stillbirths.

## Competing interests

The authors declare no conflict of interests.

## Authors’ contributions

Professor Zulfiqar A Bhutta developed the review parameters and secured support. Drs Sidra Ishaque, Yawar Yakoob and Aamer Imdad, undertook the literature search, data extraction and analysis with advice and input from Professor Robert Goldenberg, Dr Thomas Eisele and  Professor Bhutta. Professor Zulfiqar A. Bhutta gave advice on all the aspects of the project and was the overall supervisor.

## Supplementary Material

Additional File 1 A word document containing a review of literature for specific infections related to stillbirths Click here for file

Additional File 2 A word document containing the basic search strategies and terms used for specific infections. Click here for file

Additional File 3 An excel file that contains the data extraction sheet of studies included in the review Click here for file

Additional File 4 A word file that described the characteristics of studies included in the main meta-analysis Click here for file
