# Supplementary material for: Effectiveness of interventions to screen and manage infections during pregnancy on reducing stillbirths: a review
Source: BMC Public Health. 2011 Apr 13;11(Suppl 3):S3. doi: 10.1186/1471-2458-11-S3-S3 (PMC3231903; doi:10.1186/1471-2458-11-S3-S3)
Supplement: Additional File 1 — A word document containing a review of literature for specific infections related to stillbirths [file 1471-2458-11-S3-S3-S1.docx]

**Additional File 1: Review of literature for specific infections related to stillbirths**

**Syphilis**

Syphilis is caused by the spirochete Treponema pallidum. Spirochetes can cross the placenta and infect the fetus usually after 14 weeks’ gestation, with risk of fetal infection increasing with gestational age. If the fetus is infected, about 45% will die in utero, with another 30–40% born alive but with signs of congenital syphilis [73]. A pregnant woman has a 70% chance of transmitting the infection to her fetus in early untreated syphilis [74]. Stillbirths due to syphilis, despite being a major health challenge, should be easy to eradicate. Within a functioning health system, it is feasible to screen pregnant women for syphilis and once diagnosed, easy and inexpensive to treat. Testing for syphilis within antenatal clinics can be adopted as a strategy to improve treatment of syphilis and reduce perinatal mortality, since the test results are available immediately, followed by initiation of early treatment [75]. To address some of these issues, a recent study suggested that point-of-care rapid testing and treatment is the most cost-effective method to reduce adverse pregnancy outcomes associated with syphilis [76].

**Malaria**

Malaria is a major public health problem (can cite Hay S. et al 2010 PLoS Med). More than 40% of all births worldwide occur in areas where malaria is endemic [7]. Malaria is a parasitic disease spread by one of four intracellular parasites transmitted by various types of mosquito species and is endemic in parts of Africa, Asia, and South America. Approximately 50 million pregnant women are exposed to malaria each year [58]. The burden of malaria infection during pregnancy is caused mainly by the *Plasmodium falciparum* parasite. The impact of malaria on pregnancy outcomes varies based on the type of malaria, maternal immune (based on local transmission pressure) and parity. *P. falciparum* is most commonly implicated in stillbirth, but *P.vivax* has also recently been associated [7]. Pregnancy outcome is also directly related to the extent of placental malaria, reported to occur in 13-63% of maternal infections [77] [78]. Many studies, mostly from endemic areas, have found that placental malaria is associated with twice (OR = 2.19) the stillbirth risk [62]. Newman et al. reports a seven-fold increased risk of stillbirth with placental malaria in sub-Saharan African area of unstable transmission [79]. This suggests that malaria is likely to be an important cause of stillbirth, especially in populations of pregnant women experiencing a malaria infection for the first time. A Tanzanian study showed a PAF of malaria for stillbirth of 32 % [80]. Together, these findings suggest that malaria is an important preventable infectious cause of stillbirth in LMICs. Though a few studies have demonstrated increased risk for malaria-associated stillbirth in Australia and India, most malaria research has focused on Africa. Further research is needed in geographic areas with low or unstable malarial transmission, such as Asia, to better understand the global impact of malaria on stillbirth.

**HIV**

The human immunodeficiency virus (HIV) pandemic is one of the major public health crises today. Given that 13 million women of reproductive age currently live with HIV in sub-Saharan Africa [81], this is the geographic region reporting the highest incidence of stillbirth in the world [82]. HIV infection and its consequences may account for a high toll of stillbirths. Nevertheless, little is known on predictors of stillbirth among HIV-infected women [65].

**Bacterial vaginosis, asymptomatic bacteriuria and ascending infections**

***Bacterial vaginosis*** is an imbalance of vaginal flora caused by a reduction of the normal lactobacillary bacteria, and a heavy overgrowth of mixed anaerobic flora, including Gardnerella vaginalis, Mycoplasma hominis and Mobiluncus species. Bacterial vaginosis does not appear to be sexually transmitted but may be associated with sexual activity. The natural history of bacterial vaginosis is such that it may spontaneously resolve without treatment although most women identified as having bacterial vaginosis in early pregnancy are likely to have persistent infection later in pregnancy [83]. A substantial amount of evidence exists relating bacterial vaginosis in pregnancy to poor perinatal outcome, particularly, with an increased risk of preterm birth [83] [84] [85] [86] and potential neonatal sequelae due to prematurity. However, there is a dearth of studies reporting the relationship of bacterial vaginosis and stillbirths or perinatal mortality.

***Asymptomatic bacteriuria***, generally defined as true bacteriuria in the absence of specific symptoms of acute urinary tract infection, occurs in 2% to 10% of all pregnancies [87]. E. coli is the most common pathogen associated with asymptomatic bacteriuria, representing at least 80% of isolates. Other organisms include other gram-negative bacteria and group B streptococci. Screening for and treatment of asymptomatic bacteriuria in pregnancy has become a standard of obstetric care and most antenatal guidelines include routine screening for asymptomatic bacteriuria.

***Ascending bacterial infection*** is a common cause of stillbirth, but interventions geared towards both prevention and treatment have proven elusive. Organisms that ascend from the vagina into the uterus enter the amniotic fluid either through intact choriodecidual membranes or after membrane rupture [88]. The fetal lung is the most common organ infected, associated with fetal breathing of contaminated amniotic fluid.

**TORCH infections (Toxoplasmosis, Rubella, CMV and Herpes infections)**

***Toxoplasma gondii*** is a parasite that normally spends its lifecycle in animals, but may be transmitted to humans through contact with animal feces or from undercooked meat. Its human infection, toxoplasmosis, despite being a widespread parasitic disease, is generally asymptomatic or only causes mild disease, but has been identified in case reports as a cause of stillbirth [89].

***Maternal rubella*** has been implicated as a cause of stillbirths [51]. Consequences of the occurrence of rubella in early pregnancy, specially congenital cataracts were originally reported by Gregg in 1941[90]. Transmission of the virus to the fetus mostly occurs with maternal disease during the first trimester; the risk of fetal damage decreases as gestational age increases. Subsequently, other anomalies have been documented, including major cardiac defects, some resulting in stillbirth later in pregnancy. Rubella outbreaks still continue to be routinely reported from many LMICs; however, little is known regarding their contribution to stillbirths [51].

***Cytomegalovirus (CMV)*** is the most common congenital viral infection [91]. The highest rate of transmission to the fetus, and the most severe consequences, occur with primary infection, probably due to lack of transferred immunity. In many women, the CMV infection is chronic. However, despite the presence of maternal antibodies, the fetus is at some risk. Placental involvement is well-documented. The degree of fetal damage is also higher in early primary infection than in late infection. Griffiths and Baboonian [92] in a prospective study of >10, 000 women found increases in fetal loss associated with early CMV infections. It was recently suggested that serial urine studies for CMV by PCR may also be a useful tool to detect primary infection in pregnancy since CMV is secreted in the urine in most such infected women [93].Whether CMV actually causes stillbirth and, if so, the mechanism by which it does, is not clear.

***Herpes simplex*** infections have also been described as a rare cause of fetal death [94]. However, Herpes simplex viruses rarely, if ever, cause stillbirth, likely because the virus rarely causes an intrauterine infection; neonatal infections are acquired during fetal passage through an infected birth canal. There is a paucity of evidence with regards to the significance of Herpes virus in causing stillbirths. Also, whether treatment and prevention have a significant positive impact on adverse pregnancy outcomes remains widely elusive.

The relationship between pregnancy and infections has been studied for many years. Overall, little consensus exists on the true relationship between infection and stillbirth. Several important reasons have been quoted in literature. Important infections may still be missed even with available histopathology and cultures of the placenta and fetal autopsies. Positive serologic tests and organisms in the placenta or fetus may rarely show an association [95] [3] [96] [97]. More importantly, the actual infection is seldom obvious from the case history or physical examination of the mother or fetus. Finally, an infection may indirectly lead to stillbirth through a cascade of events that may preclude appreciation of its role in causing fetal death (e.g., rubella infections leading to stillbirths by causing congenital anomalies) [2]
