# Supplementary material for: Effectiveness of interventions to screen and manage infections during pregnancy on reducing stillbirths: a review
Source: BMC Public Health. 2011 Apr 13;11(Suppl 3):S3. doi: 10.1186/1471-2458-11-S3-S3 (PMC3231903; doi:10.1186/1471-2458-11-S3-S3)
Supplement: Additional File 2 — A word document containing the basic search strategies and terms used for specific infections. [file 1471-2458-11-S3-S3-S2.docx]

**Additional File 2: The basic search strategies used for specific infections are as follows**

**Syphilis**

Syphilis search was done with the TORCH infections as given below.

**Malaria**

# A combination of the following search terms was used using ‘MeSH’ and ‘All Fields’ terms: “stillbirth”, “fetus”, “fetal”, “loss”, and “malaria”.

**HIV**

("Antiretroviral Therapy, Highly Active"[Mesh] OR "HIV"[Mesh] OR HIV OR HAART OR “Highly active antiretroviral therapy”) AND (mother* OR Pregnancy OR maternal) AND (“perinatal death” OR stillbirth* OR “fetal death” OR “perinatal mortality” OR “fetal demise” OR “intrauterine death”)

**Bacterial Vaginosis**

("Bacterial vaginosis") AND ("Screening" OR "prevention" OR management OR treatment OR antibiotic*) AND ("Pregnancy"[Mesh] OR "Mothers"[Mesh] OR mother* OR pregnan* OR maternal) AND (“perinatal death” OR stillbirth* OR “fetal death*” OR “fetal mortality” OR “fetal demise” OR “intrauterine death” OR “perinatal mortality”)

**Antibiotics for chorioamnionitis**

(antibiotic*) AND (chorioamnionitis OR “intrauterine infection”) AND (stillbirth* OR "perinatal mortality" OR "perinatal death*"OR "fetal death*" OR "intrauterine death*")

**Asymptomatic bacteriuria**

("Asymptomatic bacteriuria") AND ("Screening" OR "prevention" OR management OR treatment OR antibiotic*) AND ("Pregnancy"[Mesh] OR "Mothers"[Mesh] OR mother* OR pregnan* OR maternal) AND (“perinatal death” OR stillbirth* OR “fetal death*” OR “fetal mortality” OR “fetal demise” OR “intrauterine death” OR “perinatal mortality”)

**Periodontal disease**

periodontal AND (pregnancy OR maternal) AND (stillbirth* OR "perinatal mortality" OR "fetal death*" OR "intrauterine death*" OR miscarriage)

**Helminthiasis**

(helminth OR “anti-helminthic” OR antihelminthic OR deworming) AND (pregnancy OR maternal) AND (stillbirth* OR "perinatal mortality" OR "fetal death*" OR "intrauterine death*" OR miscarriage OR abortion*)

**TORCH Infections**

(Toxoplasmosis OR Rubella OR Cytomegalovirus OR Herpes OR Syphilis OR TORCH) AND (mother* OR Pregnancy OR maternal) AND (“perinatal death” OR stillbirth* OR “fetal death” OR “perinatal mortality” OR “fetal demise” OR “intrauterine death”)
