# Supplementary material for: Effectiveness of interventions to screen and manage infections during pregnancy on reducing stillbirths: a review
Source: BMC Public Health. 2011 Apr 13;11(Suppl 3):S3. doi: 10.1186/1471-2458-11-S3-S3 (PMC3231903; doi:10.1186/1471-2458-11-S3-S3)
Supplement: Additional File 4 — A word file that described the characteristics of studies included in the main meta-analysis [file 1471-2458-11-S3-S3-S4.docx]

**Additional File 4: Characteristics of studies included in the main meta-analysis**

| **Reference** | **Country** | **Participants/ sample size** | **Intervention** | **Control** | **Design/limitations** | **Quality** |
| --- | --- | --- | --- | --- | --- | --- |
| **Syphilis** | | | | | | |
| Gichangi 2004 [98] | Kenya | 357 RPR positive women delivering in the study hospital | Penicillin regime not stated | None | Cohort/No adjusting for confounding | low |
| Greenblatt 1950 [99] | Full text not available | | | | | |
| Ingraham 1950 [100, 101] | U.S. | 965 pregnant women with syphilis attending study hospital | A total of 2.4 million units IM penicillin (or more) (different dosing regimens) | None | Cohort/No adjusting for confounding | low |
| Lu 2001 [102] | China | 64 positive women attending antenatal clinic | Penicillin regimen not stated | None | Cohort/Full paper in Chinese. No adjusting for confounding | low |
| Myer 2003 [75] | S.Africa | 1043 RPR positive women attending 1st ANC | 1 - 3 doses of 2.4million units pen G | None | Cohort/No adjusting for confounding in this analysis | low |
| Rutgers 1993 [103] | Zimbabwe | 156 women RPR positive at ANC | 1 dose of 2.4million units pen G STAT >28 days prior to delivery | None | Cohort/No adjusting for confounding | low |
| Southwick 2001 [104] | Russian Federation | 714 High risk women RPR positive | penicillin regime not stated | None | Cohort/No adjusting for confounding | low |
| Tikhonova 2003 [105] | Russian Federation | 634 women delivering in study hospital at >20/40 with a positive non-treponemal test in pregnancy | history of "penicillin treatment given" in notes | None | Retrospective Cohort/No adjusting for confounding. Rectrospective relying on documentation of treatment - some controls may have also been treated, but not recorded | Very low |
| **Malaria** | | | | | | |
| Challis 2004 [27] | Mozambique,Africa | 600  nulliparous and primiparous women under 21 years | Sulfadoxine-pyrimethamine (3 tablets): at enrolment and in third trimester | Placebo | RCT/Allocation concealment unclear | Moderate |
| Njagi 2002 [106, 107] | Kenya | 963 women in their first or second pregnancy | 1. ITNs plus sulfadoxine-pyrimethamine  2. ITNs  3. Sulfadoxine-pyrimethamine  Net size (cm): 190 wide by 180 long by 150 high  Net material: polyester | Placebo | Individual RCT/none in particular | High |
| Parise 1998 [25] | Kenya | 2077 antenatal clinic attendees; first or second pregnancy | Sulfadoxine-pyrimethamine: treatment dose, repeated in late pregnancy | No intermittent preventive treatment, sulfadoxine-pyrimethamine given | Quasi RCT/none in particular | Moderate |
| Shulman 1999 [26] | Kenya | 1264 primigravidae attending antenatal clinics at a health centre (1) or hospital (1); singleton pregnancy;  16 to 30 weeks gestation | Sulfadoxine-pyrimethamine: recruited at 16 to 19 weeks (2 doses); 20 to 26 weeks (2 doses); 27 to 30 weeks (1  dose)  Other:ferrous sulphate; impregnated bed nets in use in the area | Placebo | RCT/none in particular | High |
| Shulman 1998 [108] | Kenya | 503 women pregnant for the first time with singleton pregnancies or history of previous pregnancy that did not go  beyond 12 weeks | ITNs  Insecticide: permethrin (500 g/m2)  Size (cm): 190 wide by 180 long by 150 high | No NEts | Cluster-RCT/  small numbers ; not adjusted for clustering and it is plausible that the  women who delivered in hospital were different to those who  delivered at home, which means they are unlikely to be  representative of all those randomized.  all women were visited at home at least  four weeks (range four weeks to nine months) after their expected  date of delivery to ascertain birth outcome in terms of stillbirths,  neonatal deaths, and maternal deaths. This range means that  there maybe variability in the reliability of the data collected. | Moderate |
| ter Kuile 2003 [109] | Kenya | 2991 pregnant women of all parity | ITNs  Insecticide: permethrin; pretreated at distribution and re-treated biannually to maintain a target dose of 500 mg/m2 | No nets | Cluster-RCT/  results were presented for women with no to three  previous pregnancies, and those with four or more previous  pregnancies. The results for the first group were combined with  subgroups considering women in their first or second pregnancy. This means that  stratification by gravidity could not always be presented as  desired. | Moderate |
| **HIV** | | | | | | |
| DITRAME[28] | Cote d’Ivoire and Burkina  Faso | 431 women aged 18+ years positive for HIV-1 or both HIV-1 and HIV-2 who presented before 32 weeks  gestation who lived in and planned to give birth in the area. | ZDV arm: MOTHER 300mg twice daily from 36-38 weeks until onset of labour; 600 mg at start of  labour and 300mg twice daily until 7 days after birth. | Placebo | RCT-none in particular | High |
| Limpongsanurak 2001 [29] | Thailand | 182 HIV-1 positive women aged 15 to 40 years at 37 weeks gestation with no history of ARV use, no intention  to breastfeed, no fetal abnormality, haemoglobin >8g/dl and willing to bring child for follow up at 6  months after delivery. | ZDV arm:MOTHER - ZDV 250mg orally twice daily from38 weeks gestation until onset of labour then  ZDV intravenously at 2mg/kg for first hour of labour followed by 1mg/kg/hr until delivery. INFANTS -  no treatment | Placebo: Identical placebo capsules and 5% intravenous dextrose in half strength normal saline in  intrapartum period at same dosing schedule. | RCT/ Small number of participants,Allocation concealment: Unclear. | Moderate |
| PACTG 076 [33] | USA and France | 477 HIV +ve women at 14-34 weeks gestation with CD4 count >200 | ZDV arm. MOTHERS: ZDV 100mg orally x 5 per day from time of presentation (14 - 34 weeks) until  onset of labour; intravenous ZDV 2mg/kg loading dose over one hour follwed by 1mg/kg/h until delivery.  INFANTS: ZDV syrup 2mg/kg six hourly for six weeks, beginning 8 - 12 hrs after birth | Placebo | RCT/ Unclear Allocation concealment;Blinding unclear | Moderate |
| PETRA [32] | South Africa,  Tanzania and Uganda | 1457( A 366, B 371,  C 368, PL 352)a HIV-1 +ve women with gestational age <36 weeks, age > 18 years or legal age of consent, Hb>8g/L and  18 months follow up possible. | **Arm A:**MOTHERS: Oral zidovudine (ZDV) plus Lamivudine (3TC) from 36 weeks gestation until 7  days after delivery. ZDV 300mg/3TC 150mg twice daily from 36 weeks, ZDV 300mg/3TC 150mg at  onset of labour, ZDV 300mg 3 hourly and 3TC 150mg 12 hourly during labour and ZDV 300mg/3TC  150mg twice dail for 7 days postpartum. INFANTS: ZDV 4mg/kg plus 3TC 2mg/kg twice daily for first  7 days after birth  **Arm B:**MOTHERS: Oral zidovudine (ZDV) plus Lamivudine (3TC) from the start of labour until 7  days after delivery.Dosing schedule the same as for ArmA except for the loading dose at the start of labour  of ZDV 600mg/3TC 150mg. INFANTS: ZDV plus 3TC for first 7 days after birth as for Arm A.  **Arm C:**MOTHERS: ZDV plus 3TC during labour only. ZDV 600mg/3TC 150mg at onset of labour  followed by ZDV 300mg 3 hourly and 3TC 150mg 12 hourly until delivery. INFANTS: None  **Co-interventions -**MOTHERS:Multivitamins postnatally; INFANTS: Cotrimoxazole prophylaxis up to  months after birth | Placebo | RCT/none in particular | High |
| RETRO-CI [31] | Abijan,Coˆ te d’Ivoire | 280 HIV-1 positive women at 36 weeks gestation who were 18+ years old | ZDV arm:MOTHER -Oral zidovudine 300mg twice daily from36 weeks gestation until onset of labour,  300mg at onset of labour then 300mg every 3 hours until delivery. INFANT - no treatment | Placebo | RCT/none in particular | High |
| Thai-CDC [30] | Thailand | 423 women who were HIV-1 +ve within 28 days before randomisation, were > 18 years at <= 34 weeks  gestation | Oral zidovudine 300mg twice daily from 36 weeks until onset of labour and taken once at  onset of labour and then 300mg every 3 hours until delivery | Placebo | RCT/none in particular | High |
| **Bacterial vaginosis** | | | | | | |
| Kekki 2001 [110] | Finland | Pregnant women with BV (screened at 10-17 weeks, using Spiegel’s criteria). | 2% vaginal clindamycin cream (single course) for 7 days. Randomized @  12-19 weeks. | Placebo | RCT/none in particular | High |
| Lamont 2003 [37] | Not mentioned | Asymptomatic pregnant women 13-20 weeks with BV or intermediate flora by Nugent’s criteria | 5 g of 2% clindamycin intravaginal cream (+ 100 mg) or placebo for 3 nights. In addition 7 extra days if  vaginal swab still positive (BV/intermediate flora) at visit 2. | None | RCT/Intent-to-treat analysis. 30 did not return for visit 2 in clindamycin group, and 11 in the placebo group,  leaving 208 antibiotic vs 201 placebo.Allocation concealment unclear. | Moderate |
| McDonald 1997 [34] | Not mentioned | Pregnant women at 18 weeks’ gestation with BV or Gardnerella vaginalis. | Metronidazole 400 mg x 2/day for 2 days at 24 weeks’ gestation | Placebo | RCT/none in particular | High |
| NICHD MFMU 2000 [35] | Not mentioned | Pregnant women at 16-23 + 6 weeks with asymptomatic BV (not TV+) (screened at 8-22 + 6 weeks)  gestation. | 8 x 250 mg dose oralmetronidazole or placebo plus repeat dose in 48 hours (@ 16-23 + 6 weeks’ gestation)  .  Second treatment at 24-30 weeks’ gestation. | None | RCT/Low recruitment response - only 29% BV+ women were enrolled. 10% did not return for follow up  visit, leaving 953 antibiotic vs 966 placebo. Unpublished data on neonatal morbidity and admission to a  neonatal unit were supplied by the authors.No allocation concealment | Moderate |
| Odendaal 2002 [36] | Not mentioned | 2 groups of women with BV (Spiegel’s criteria): primigravidae at first antenatal visit, between 15 and 26  weeks’ gestation; women with a previous preterm labour/midtrimester miscarriage | Oral metronidazole 400 mg twice daily for 2 days and if still BV positive after 4 weeks, repeat treatment  course | Placebo:100 mg vitamin C | RCT/  Lost to follow up participants not separated into treatment/placebo. Intention-to-treat analysis of  128 antibiotic vs 127 placebo. | Moderate |
